# A new nutritional risk index for predicting mortality in hemodialysis patients: Nationwide cohort study

**DOI:** 10.1371/journal.pone.0214524

**Published:** 2019-03-28

**Authors:** Eiichiro Kanda, Akihiko Kato, Ikuto Masakane, Yoshihiko Kanno

**Affiliations:** 1 Medical Science, Kawasaki Medical School, Kurashiki, Okayama, Japan; 2 Blood Purification Unit, Hamamatsu University Hospital, Hamamatsu, Shizuoka, Japan; 3 Department of Nephrology, Honcho Yabuki Clinic, Yamagata, Yamagata, Japan; 4 Department of Nephrology, Tokyo Medical University, Shinjuku, Tokyo, Japan; Hopital Europeen Georges Pompidou, FRANCE

## Abstract

**Background:**

Protein energy wasting (PEW) is a risk factor for death. However, the cutoff vales for PEW are not optimized for early identification of hemodialysis patients with malnutrition. We evaluated the prognosis of Japanese maintenance hemodialysis patients using nutritional indices optimized for them.

**Materials and methods:**

We analyzed data from a nation-wide prospective cohort study of the Japanese Society for Dialysis Therapy Renal Data Registry to develop and validate a nutritional risk index (n = 48349, 48349, respectively). The association of nutritional factors with one-year death was tested using Cox proportional hazards models. Their cutoff levels were determined from the hazard ratios or receiver operating characteristic curves. Then, risk index was developed using scoring models.

**Results:**

Male was 61.4%; average age, 65.7±12.2 years; and diabetes mellitus, 32.8%. Four clinical factors were retained in the final model: low BMI (<20kg/m^2^), yes = 3, no = 0; low serum albumin level (young <3.7g/dL; old <3.5g/dL), yes = 4, no = 0; abnormal serum total cholesterol level, low (<130mg/dL) = 1, high (220≥mg/dL) = 2, no = 0; low serum creatinine level (young female, <9.7mg/dL; old female, <8.0mg/dL; young male, <11.6mg/dL; old male, <9.7mg/dL), yes = 4, no = 0. In the validation dataset, medium- and high-risk groups (total score 8 to 10; 11 or more) showed a higher risk of all-cause death than the low-risk group (0 to 7): medium-risk group (10.5%), hazard ratio adjusted for baseline characteristics 1.96 (95% confidence interval 1.77, 2.16); high-risk group (8.2%), 3.91 (3.57, 4.29). The medium- and high-risk groups also showed a higher risk of cardiovascular disease- and infection-caused deaths than the low-risk group.

**Conclusion:**

We developed a new nutritional risk index for hemodialysis patients, which may detect patients with malnutrition with a high-risk of death.

## Introduction

High rates of malnutrition in dialysis patients have been reported[1–3]. Protein energy wasting (PEW) is the state of decreased body stores of protein and fat mass, and is often associated with mortality, comorbid conditions, and reduced activities of daily living[4]. To improve their prognosis, early identification of patients with PEW and interventional treatments for their nutritional conditions are necessary[5].

The International Society of Renal Nutrition and Metabolism proposed the criteria for PEW[4]. The criteria include a serum albumin level of less than 3.8g/dL and a serum cholesterol level of less than 100mg/dL. According to the Annual Dialysis Data Report 2014 of the Japanese Society for Dialysis Therapy (JSDT) Renal Data Registry (JRDR), which is a nationwide renal data registry and contains data of all dialysis patients (n = 358775) in Japan, the mean serum albumin and cholesterol levels in dialysis patients were 3.60±0.44g/dL and 154.7±35.3mg/dL, respectively[6]. Assuming that these nutritional factors show normal distributions, it can be estimated that 67.5% of the patients had serum albumin levels of less than 3.8g/dL and 6.1% had serum cholesterol levels of less than 100mg/dL. The difference in the distribution of the patients between the two criteria suggests that the patients may be differently diagnosed as having PEW on the basis of each criterion. Therefore, reference values of the criteria for PEW diagnosis of hemodialysis patients are required.

For the early identification and treatment for patients with malnutrition, multiple evaluation of their nutritional condition by various nutritional assessments including anthropometric, biochemical/biophysical, clinical, and dietary methods are needed. However, it is very difficult to evaluate nutritional conditions of all hemodialysis patients using these methods. At clinical settings, high-risk patients are screened first, then their nutritional conditions are thoroughly evaluated, and finally, malnutrition is diagnosed. Therefore, the aims of this study were to develop a nutritional risk index for hemodialysis patients (NRI) for screening hemodialysis patients with high risk of death on the basis of the concept of PEW using JRDR data, and to evaluate the prognosis of these patients. (1) After the identification of the main risk factors for one-year death using JRDR data of hemodialysis patients, a new risk index was developed. (2) The accuracy of the index to predict death was also evaluated.

## Materials and methods

### Dataset

This is a prospective cohort study of maintenance hemodialysis patients using JRDR data. JSDT has been conducting annual surveys of dialysis facilities in Japan since 1968. The JRDR data from 2008 to 2009 were used in this study. This study was approved by the ethics committee of JSDT and was exempt from the need to obtain informed consent from participants (JSDT No. 6). The data were analyzed anonymously. The study was performed in accordance with the relevant guidelines and the Declaration of Helsinki of 1975 as revised in 1983.

**Fig 1:**
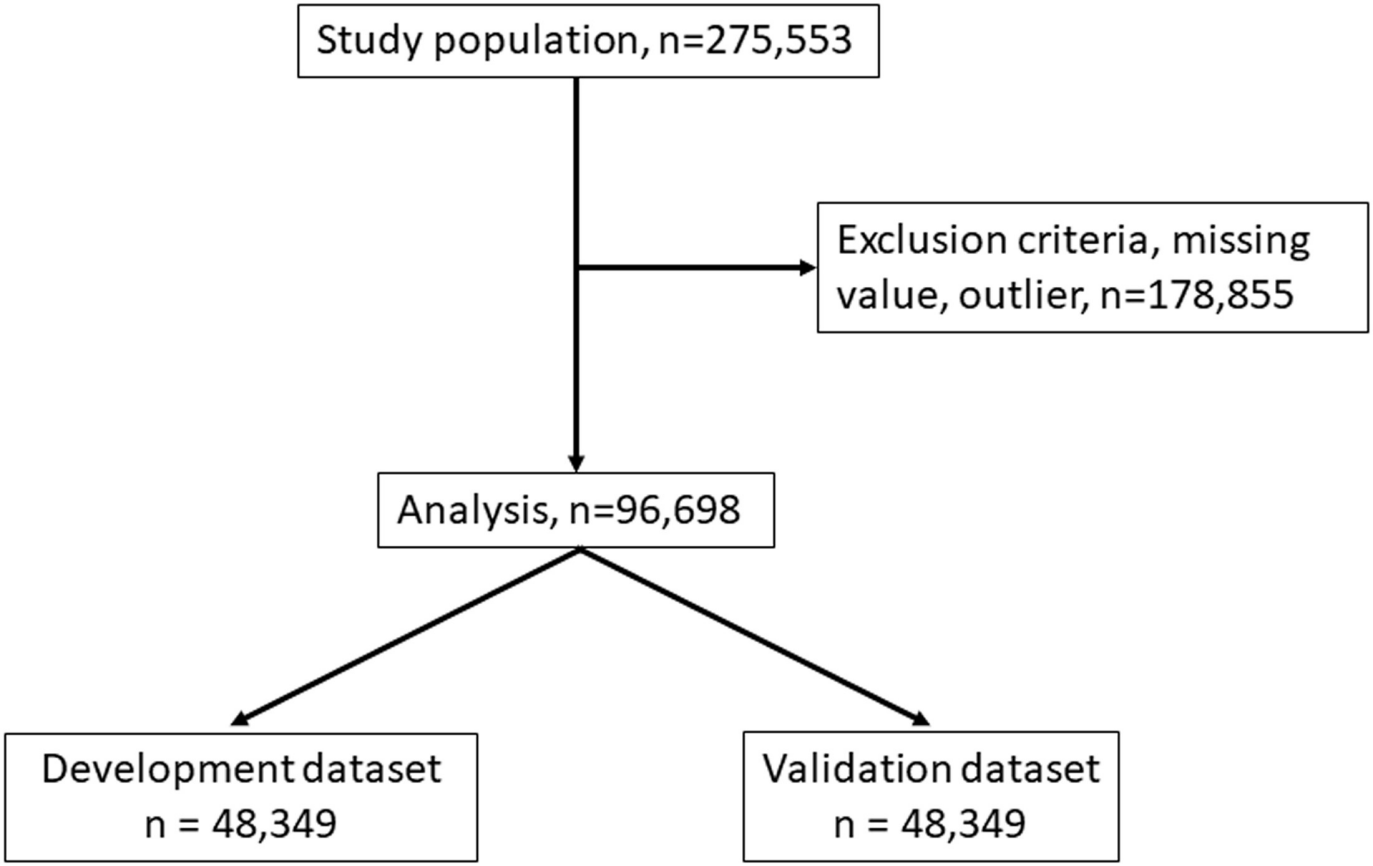
Randomization of study population and development of datasets.

The subjects of this study were the 275553 patients (Fig 1).

The sample size was evaluated by alpha error and power. The exclusion criteria were as follows: patients younger than twenty years; patients on hemodiafiltration, hemofiltration, or peritoneal dialysis; patients with missing values or outlier values of laboratory data; patients who had a limb amputated; and patients with a hemodialysis vintage of less than one year. Thus, 96698 subjects were included in the analysis. The included subjects were randomly classified into two groups to obtain (1) a dataset for the development of NRI (development dataset, 48349) and (2) a dataset for validation of NRI (validation dataset, 48349). The sample size was evaluated to maximize statistical power.

The baseline data were as follows: gender; age; history of cardiovascular disease (CVD); diabetes mellitus (DM) as a cause of end stage renal disease (ESRD); vintage; body mass index (BMI); serum albumin, total cholesterol, creatinine, phosphorus, and C reactive protein (CRP) levels; hemoglobin level; normalized protein catabolic rate (nPCR) and Kt/V. The laboratory data were measured before hemodialysis, and BMI was measured on the basis of weight measured after hemodialysis. The outcome was death including all-cause death and CVD- and infection-caused deaths within one year.

### Statistical analyses

Normally distributed variables are presented as mean±standard deviation; otherwise, the median and interquartile ranges are presented. Highly skewed variables were transformed with the natural logarithm function prior to use in models [ln(vintage), ln(CRP)]. Intergroup comparisons of parameters were performed using the chi-square test, t-test, and Mann-Whitney U test as appropriate after F-test of equality of variances between the groups. Subjects were categorized on the basis of age (young, less than 65 years; old, 65 years or older) and on the basis of CRP (absence of inflammation, less than 1.0mg/dL; presence of inflammation, 1.0mg/dL or higher).

Step 1: (1) In the development dataset, cutoff levels of BMI; serum albumin, total cholesterol, and creatinine levels; and nPCR were determined. To control confounders, multivariate models were examined following analysis. A multivariate Cox proportional hazards model was used to evaluate the relationships between risks of all-cause death and the nutritional factors, namely, serum albumin, total cholesterol, and creatinine levels; BMI; and nPCR, using the splines of these nutritional factors. This multivariate Cox proportional hazards model was adjusted for baseline characteristics such as gender, age, CVD, DM, ln(vintage), serum phosphorus level, ln(CRP), hemoglobin level, and KT/V. If the relationship between the nutritional factors and the risks of all-cause death were not linear, the cutoff level was determined on the basis of the relationship. If the relationship was linear, the cutoff level was determined on the basis of the area under a receiver operating characteristic (ROC) curve (AUC) for the prediction of all-cause death with Youden index and baseline characteristics such as age, gender, and DM. Then, the nutritional factors were categorized into two or three groups on the basis of the cutoff levels, and scored from 0 to 1 or 2. (2) To develop a risk scoring model, a Cox proportional hazards model was constructed including the categorical nutritional factors and baseline characteristics. A weighted score proportional to the smallest parameter estimate of the nutritional factor was assigned to each nutritional index, which was rounded to the nearest integer. For each subject, the risk score was calculated using the risk scoring model as the sum of the points. (3) On the basis of categorical criteria for the risk scoring model, the subjects were divided into three risk groups using Kaplan-Meier survival curves (low-, medium- and high-risk groups). Then, the survival curves of the risk groups were evaluated on the basis of Kaplan-Meier survival curves. Cox proportional hazards models adjusted for the baseline characteristics were used to compare the risk of an outcome between the risk groups. The results are presented here as hazard ratios (HRs) with 95% confidence interval (CI).

Step 2: Risk score was calculated for each subject using the validation dataset. On the basis of the categorical criteria for the risk score, subjects were divided into three risk groups. Subjects’ survival curves were evaluated by Kaplan-Meier analysis. The risk of the outcome was compared between the risk groups using Cox proportional hazards models adjusted for baseline characteristics. In the analysis of competing risks of cause-specific death, Fine and Gray competing risk regression models adjusted for the baseline characteristics were also examined. Then, subgroup analysis on the basis of age, gender, DM and inflammation, was carried out to evaluate the relationships between the risks of all-cause death and the risk groups using adjusted Cox proportional hazards models. These analyses were conducted using SAS version 9.4 (SAS, Inc., NC, USA) and R version 3.4.1 (R project for Statistical Computing, Vienna, Austria). Statistical significance was defined as *p* < 0.05.

## Results

### Cutoff levels of nutritional indices

The baseline characteristics including biochemical data are shown in Table 1.

**Table 1:** Baseline characteristics.

	All	Development dataset	Validation dataset	*p*
N	96698	48349	48349	
Male (%)	59418 (61.4)	29750 (61.5)	29668 (61.3)	0.59
Age (years)	65.7±12.2	65.7±12.2	65.7±12.2	0.79
CVD (%)	18425 (19.1)	9129 (18.9)	9296 (19.2)	0.17
DM (%)	31686 (32.8)	15791 (32.7)	15895 (32.9)	0.48
Vintage (years)	8.3±6.7 6.2 (3.3, 11.1)	8.3±6.7 6.2 (3.3, 11.1)	8.3±6.7 6.2 (3.3, 11.1)	0.44
BMI (kg/m^2^)	22.1±3.5	21.2±3.4	21.1±3.4	0.55
Albumin (g/dL)	3.7±0.4	3.7±0.4	3.7±0.4	0.38
Creatinine (mg/dL)	10.7±2.8	10.7±2.8	10.7±2.8	0.14
Total cholesterol (mg/dL)	153.7±34.6	153.7±34.6	153.6±34.5	0.75
Phosphorus (mg/dL)	5.3±1.4	5.3±1.4	5.3±1.4	0.33
CRP (mg/dL)	0.49±1.41 0.11 (0.05, 0.34)	0.49±1.38 0.11 (0.05, 0.34)	0.49±1.44 0.11 (0.05, 0.34)	0.52
Hemoglobin level (g/dL)	10.4±1.2	10.4±1.2	10.4±1.2	0.52
nPCR	0.88±0.17	0.88±0.17	0.88±0.18	0.14
Kt/V	1.41±0.28	1.41±0.28	1.41±0.28	0.71
All-cause death (%)	6442 (6.7)	3260 (6.7)	3182 (6.6)	0.32
CVD-caused death (%)	2736 (2.8)	1364 (2.8)	1372 (2.8)	0.89
Infection-caused death (%)	1173 (1.2)	601 (1.2)	572 (1.2)	0.41

Variables are expressed as mean±standard deviation. Vintage and CRP are also shown as median and interquartile range. Intergroup comparisons of parameters were performed using the chi-square test, t-test, and Mann-Whitney U test as appropriate.

Abbreviations: CVD, cardiovascular disease; DM, diabetes mellitus as a cause of end-stage renal disease; BMI, body mass index; CRP, C reactive protein; nPCR, normalized protein catabolic rate.

**Fig 2:**
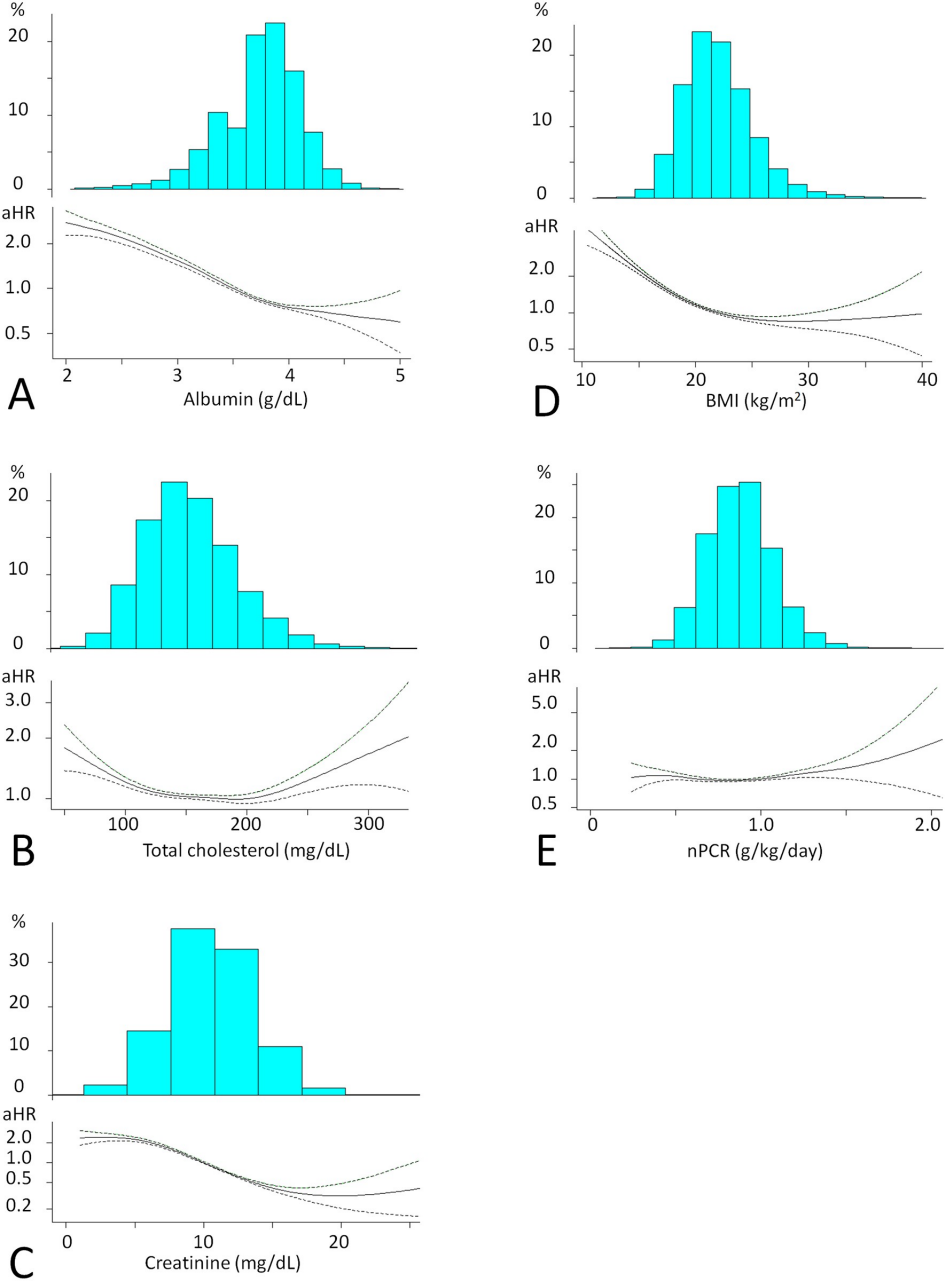
Nutritional factors and risks of all-cause death. Histogram of each nutritional factor and the aHR of all-cause death are shown in the upper and lower panels, respectively. A Serum albumin level. B Serum total cholesterol level. C Serum creatinine level. D BMI. E nPCR. Abbreviations: aHR, hazard ratio adjusted for baseline characteristics; albumin, serum albumin level; total cholesterol, serum cholesterol level; creatinine, serum creatinine level; BMI, body mass index; nPCR, normalized protein catabolic rate.

The decrease in serum albumin level was associated with an increase in the risk of all-cause death (Fig 2A). The cutoff serum albumin level for all subjects was 3.6g/dL (Table 2).

**Table 2:** Cutoff serum albumin levels for prediction of death.

	AUC	Cutoff level (g/dL)	Sensitivity, specificity
All	0.714 (0.704, 0.724)	3.6	0.682, 0.630
Young	0.673 (0.650, 0.696)	3.7	0.611, 0.646
Old	0.687 (0.675, 0.698)	3.5	0.631, 0.641
Non DM	0.729 (0.717, 0.741)	3.6	0.694, 0.639
DM	0.689 (0.672, 0.705)	3.6	0.664, 0.612
Female	0.722 (0.706, 0.738)	3.5	0.631, 0.709
Male	0.711 (0.699, 0.723)	3.6	0.663, 0.648

AUCs are expressed as values with 95% CIs.

Abbreviations: AUC, area under a receiver operating characteristic curve for the prediction of all-cause death; CI, confidence interval; Young, less than 65 years; Old, 65 years or older; DM, diabetes mellitus as a cause of end-stage renal disease.

The cutoff serum albumin level of the young group differed from that of the old group. The cutoff serum albumin levels for the risk score were determined as 3.7g/dL for the young group, and 3.5g/dL for the old group. The number of subjects with a serum albumin level of less than 3.7g/dL in the young group was 5625 (25.8%), and the subjects with a serum albumin level of less than 3.5g/dL in the old group was 7529 (28.3%). The subjects with serum albumin levels of less than 3.8 and 4.0g/dL were 24234 (50.1%) and 35109 (72.6%), respectively.

The relationship between serum total cholesterol level and a risk of all-cause death shows a U-shaped curve (Fig 2B). Low risk was observed in the subjects with serum creatinine levels between 130 and 220mg/dL, and the decrease and increase in serum total cholesterol level were associated with an increase in the risk. The number of subjects with a serum total cholesterol level of less than 130mg/dL was 12150 (25.1%), that with 220mg/dL or more was 1951 (4.0%), and that with less than 100mg/dL was 1859 (3.8%).

The decrease in serum creatinine level was associated with an increase in the risk (Fig 2C). The cutoff serum creatinine level differed between the subgroups (Table 3).

**Table 3:** Cutoff serum creatinine levels for prediction of death.

	AUC	Cutoff level (mg/dL)	Sensitivity, specificity
All	0.722 (0.713, 0.731)	9.7	0.677, 0.652
Young	0.684 (0.662, 0.706)	11.0	0.648, 0.641
Old	0.678 (0.667, 0.689)	8.9	0.609, 0.649
Non DM	0.750 (0.739, 0.761)	9.8	0.689, 0.685
DM	0.676 (0.661, 0.691)	8.9	0.595, 0.670
Female	0.762 (0.748, 0.776)	8.7	0.722, 0.674
Male	0.734 (0.723, 0.745)	10.4	0.705, 0.658
Female & Young	0.720 (0.682, 0.758)	9.7	0.653, 0.696
Female & Old	0.707 (0.689, 0.724)	8.0	0.634, 0.678
Male & Young	0.700 (0.675, 0.725)	11.6	0.623, 0.671
Male & Old	0.686 (0.673, 0.700)	9.7	0.650, 0.633

AUCs are expressed as values with 95% CIs.

Abbreviations: AUC, area under a receiver operating characteristic curve for the prediction of all-cause death; CI, confidence interval; Young, less than 65 years; Old, 65 years or older; DM, diabetes mellitus as a cause of end-stage renal disease.

The cutoff levels for the risk score were as follows: the female and young group were 9.7mg/dL (2463, 31.2%); the female and old group, 8.0mg/dL (3574, 33.4%); the male and young group, 11.6mg/dL (4515, 32.5%); the male and old group, 9.7mg/dL (6282, 39.4%).

Regarding, the relationship between BMI and a risk of all-cause death shows that the decrease in BMI was associated with an increase in the risk (Fig 2D). There was no apparent difference in the cutoff BMI between the subgroups (Table 4).

**Table 4:** Cutoff BMIs for prediction of death.

	AUC	Cutoff level (kg/m^2^)	Sensitivity, specificity
All	0.632 (0.622, 0.642)	20.0	0.560, 0.629
Young	0.579 (0.556, 0.603)	20.5	0.525, 0.589
Old	0.629 (0.617, 0.641)	19.6	0.558, 0.636
Non DM	0.643 (0.630, 0.656)	19.6	0.585, 0.622
DM	0.638 (0.622, 0.654)	20.5	0.547, 0.655
Female	0.619 (0.602, 0.637)	19.5	0.574, 0.595
Male	0.646 (0.633, 0.658)	20.4	0.598, 0.612

AUCs are expressed as values with 95% CIs.

Abbreviations: AUC, area under a receiver operating characteristic curve for the prediction of all-cause death; CI, confidence interval; Young, less than 65 years; Old, 65 years or older; DM, diabetes mellitus as a cause of end-stage renal disease.

The numbers of subjects with BMIs of less than 18.5 kg/m^2^, 20.0kg/m^2^ and 23.0kg/m^2^ were 10436 (21.6%), 19153 (39.6%) and 35888 (74.2%), respectively. Considering the usefulness of the cutoff BMI, the cutoff BMI for the risk score was determined to be 20.0kg/m^2^.

NPCR was evaluated as an index of dietary protein intake. However, the relationship between nPCR and the risk of death was not shown clearly (Fig 2E). Therefore, nPCR was not included in the risk score.

### Development and categorization of risk score

A Cox proportional hazards model was developed for the evaluation of the parameter estimates of the nutritional indices, and a score was given to each index (Table 5).

**Table 5:** Parameter estimates in the initial model and risk score.

	Parameter estimate	Ratio	Score
Low BMI (≤20kg/m^2^)	0.51798	3.255279035	3
Low serum albumin level (age <65, <3.7g/dL; age ≥65, <3.5g/dL)	0.68025	4.275075415	4
Low serum total cholesterol level (<130mg/dL)	0.15912	1	1
High serum total cholesterol level (≥220mg/dL)	0.24819	1.559766214	2
Low serum creatinine level (age <65, male<11.6mg/dL, female <9.7mg/dL; age ≥65, male<9.7mg/dL, female<8.0mg/dL)	0.65957	4.145110608	4

Each parameter estimate in a Cox proportional hazards model adjusted for baseline characteristics was compared with the smallest parameter estimate (low serum total cholesterol level). Then, the risk scores were determined.

Abbreviations: BMI, body mass index.

Riskscore=lowBMI+lowserumalbuminlevel+abnormalserumtotalcholesterollevel+lowserumcreatininelevel

Low BMI, yes = 3, no = 0; low serum albumin level, yes = 4, no = 0; abnormal serum total cholesterol level, low = 1, high = 2, no = 0; low serum creatinine level, yes = 4, no = 0.

**Fig 3:**
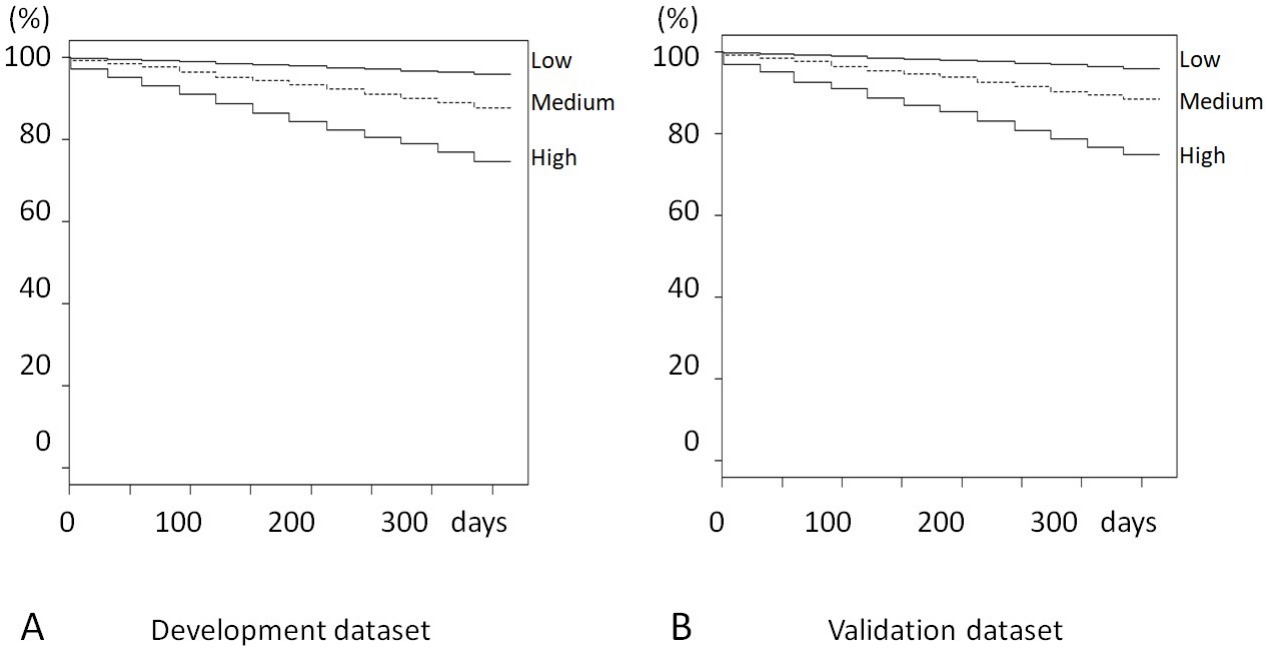
Association between risk groups and mortality. A Development dataset. B Validation dataset. Kaplan-Meier survival curves showed that the low-risk group had the highest survival probability in the both datasets. Abbreviations: Low, low-risk group; Medium, medium-risk group; High, high-risk group.

The Kaplan-Meier survival curves showed a significant difference in the survival probability of the subjects determined on the basis of risk score (log-rank test *p* = 0.0001). The subjects were categorized into three groups on the basis of the risk score: low-risk group, risk score = 0 to 7; medium-risk group, score = 8 to 10; high-risk group, score = 11 and higher. The Kaplan-Meier survival curve showed the difference in the survival probability of the groups (log-rank test *p* = 0.0001; Fig 3A). The medium- and high-risk groups showed higher risks of all-cause death than the low-risk group: the medium-risk group, HR 3.04 95% CI (2.78, 3.33), adjusted HR 1.95 95% CI (1.77, 2.15); the high-risk group, HR 6.73 95% CI (6.21, 7.29), adjusted HR 3.66 95% CI (3.33, 4.01).

### Validation of risk score and its categories

Using the validation dataset, we compared the baseline characteristics and the risk of death between the risk groups (Table 6).

**Table 6:** Baseline characteristics of the risk groups in the validation dataset.

	Low-risk group (Score 0~7)	Medium-risk group (Score 8~10)	High-risk group (Score 11~13)	*p*
N (%)	39344 (81.3)	5060 (10.5)	3945 (8.2)	
Male (%)	25697 (65.3)	2297 (45.4)	1674 (42.4)	0.0001
Age (years)	64.2±12.1	71.2±10.4	73.8±9.7	0.0001
CVD (%)	6943 (17.6)	1161 (22.9)	1192 (30.2)	0.0001
DM (%)	12620 (32.1)	1961 (38.8)	1314 (33.3)	0.0001
Vintage (years)	8.3±6.6 6.3 (3.3, 11.2)	7.7±6.9 5.3 (2.8, 10.0)	8.2±7.2 5.8 (3.0, 10.8)	0.0001
BMI (kg/m^2^)	21.6±3.3	20.8±3.3	17.4±1.7	0.0001
Albumin (g/dL)	3.8±0.3	3.4±0.4	3.1±0.3	0.0001
Creatinine (mg/dL)	11.3±2.6	8.6±2.1	7.3±1.8	0.0001
Total cholesterol (mg/dL)	155.2±33.8	146.3±43.9	147.5±35.8	0.0001
Phosphorus (mg/dL)	5.4±1.3	4.8±1.3	4.5±1.4	0.0001
CRP (mg/dL)	0.38±1.18 0.1 (0.05, 0.30)	0.83±1.98 0.2 (0.08, 0.70)	1.18±2.37 0.3 (0.10, 1.16)	0.0001
Hemoglobin level (g/dL)	10.5±1.2	10.1±1.3	9.9±1.3	0.0001
nPCR	0.90±0.17	0.82±0.18	0.80±0.19	0.0001
Kt/V	1.40±0.27	1.42±0.30	1.46±0.32	0.0001
All-cause death (%)	1602 (4.1)	584 (11.5)	996 (25.2)	0.0001
CVD-caused death (%)	743 (1.9)	233 (4.6)	396 (10.0)	0.0001
Infection-caused death (%)	215 (0.5)	127 (2.5)	230 (5.8)	0.0001

Variables are expressed as mean±standard deviation. Vintage and CRP are also shown as median and interquartile range. Intergroup comparisons of parameters were performed using the chi-square test, t-test, and Mann-Whitney U test as appropriate.

Abbreviations: CVD, cardiovascular disease; DM, diabetes mellitus as a cause of end-stage renal disease; BMI, body mass index; CRP, C reactive protein; nPCR, normalized protein catabolic rate.

Most of the subjects were categorized into the low-risk group. The baseline characteristics such as BMI; serum albumin, creatinine, total cholesterol, and phosphorus levels; hemoglobin level; and nPCR in the low-risk group tended to be higher than in the high-risk group. Age, CVD, DM as a cause of ESRD, and Kt/V were lower in the low-risk group than in the other groups. The risk of all-cause death in the high-risk group was higher than those in the other groups. The similar trends were observed in the risks of CVD- and infection-caused deaths.

The Kaplan-Meier survival curves for all-cause, CVD-caused, and infection-caused deaths showed a higher survival probability in the high-risk group than in the other groups (Fig 3B).

Cox proportional hazards models showed that the risks of all-cause, CVD-caused, and infection-caused deaths in the medium- and high-risk groups were higher than those in the low risk group (Table 7).

**Table 7:** Risk groups and risk of all-cause death.

	HR	aHR
Low-risk group	Reference	Reference
Medium-risk group	2.94 (2.68, 3.24)	1.96 (1.77, 2.16)
High-risk group	6.99 (6.45, 7.56)	3.91 (3.57, 4.29)

Values are HRs with 95% CIs of medium- and high-risk groups compared with the low-risk group.

Abbreviations: aHR, adjusted hazard ratio; CI, confidence interval.

The competing risk regression models also showed that the risks of CVD- and infection-caused deaths in the medium- and high-risk groups were higher than those in the low-risk group (Tables 8 and 9).

**Table 8:** Risk groups and risk of CVD-caused death.

	Cox proportional hazards model		Competing risk regression model	
	HR	aHR	HR	aHR
Low-risk group	Reference	Reference	Reference	Reference
Medium-risk group	2.53 (2.19, 2.93)	1.77 (1.51, 2.07)	2.47 (2.13, 2.86)	1.75 (1.49, 2.05)
High-risk group	5.99 (5.30, 6.76)	3.73 (3.24, 4.29)	5.55 (4.91, 6.27)	3.44 (2.96, 4.0)

Values are HRs with 95% CIs of medium- and high-risk groups compared with the low-risk group.

Abbreviations: CVD, cardiovascular disease; aHR, adjusted hazard ratio; CI, confidence interval.

**Table 9:** Risk groups and risk of infection-caused death.

	Cox proportional hazards model		Competing risk regression model	
	HR	aHR	HR	aHR
Low-risk group	Reference	Reference	Reference	Reference
Medium-risk group	4.77 (3.83, 5.94)	2.84 (2.25, 3.59)	4.63 (3.72, 5.77)	2.80 (2.21, 3.54)
High-risk group	12.0 (10.0, 14.5)	5.56 (449, 6.89)	10.96 (9.10, 13.20)	5.02 (4.0, 6.30)

Values are HRs with 95% CIs of medium- and high-risk groups compared with the low-risk group.

Abbreviations: aHR, adjusted hazard ratio; CI, confidence interval.

Moreover, in the subgroups based on age, gender, DM, and inflammation, the risks of all-cause death in the medium- and high-risk groups were higher than those in the low-risk group (Tables 10–13).

**Table 10:** Risk groups and risk of death in subgroups of age.

	Young		Old	
	HR	aHR	HR	aHR
Low-risk group	Reference	Reference	Reference	Reference
Medium-risk group	3.39 (2.72, 4.23)	2.62 (2.06, 3.32)	2.27 (2.04, 2.52)	1.82 (1.63, 2.04)
High-risk group	8.01 (6.55, 9.80)	6.11 (4.82, 7.75)	5.18 (4.75, 5.66)	3.59 (3.25, 3.96)

Values are HRs with 95% CIs of medium- and high-risk groups compared with the low-risk group.

Abbreviations: aHR, adjusted hazard ratio; CI, confidence interval; Young, less than 65 years; Old, 65 years or older.

**Table 11:** Risk groups and risk of death in subgroups of gender.

	Female		Male	
	HR	aHR	HR	aHR
Low-risk group	Reference	Reference	Reference	Reference
Medium-risk group	2.84 (2.42, 3.33)	1.73 (1.46, 2.04)	3.46 (3.07, 3.90)	2.12 (1.87, 2.41)
High-risk group	6.60 (5.78, 7.54)	3.59 (3.10, 4.15)	8.74 (7.91, 9.67)	4.15 (3.69, 4.67)

Values are HRs with 95% CIs of medium- and high-risk groups compared with the low-risk group.

Abbreviations: aHR, adjusted hazard ratio; CI, confidence interval.

**Table 12:** Risk groups and risk of death in subgroups of DM.

	Non-DM		DM	
	HR	aHR	HR	aHR
Low-risk group	Reference	Reference	Reference	Reference
Medium-risk group	3.06 (2.70, 3.47)	1.86 (1.63, 2.13)	2.67 (2.31, 3.09)	2.09 (1.79, 2.45)
High-risk group	7.29 (6.60, 8.07)	3.59 (3.19, 4.04)	6.48 (5.70, 7.36)	4.47 (3.86, 5.18)

Values are HRs with 95% CIs of medium- and high-risk groups compared with the low-risk group.

Abbreviations: aHR, adjusted hazard ratio; CI, confidence interval; DM, diabetes mellitus as a cause of end-stage renal disease.

**Table 13:** Risk groups and risk of death in subgroups of inflammation.

	Absence of inflammation		Presence of inflammation	
	HR	aHR	HR	aHR
Low-risk group	Reference	Reference	Reference	Reference
Medium-risk group	2.71 (2.41, 3.04)	2.01 (1.78, 2.27)	2.07 (1.74, 2.46)	1.73 (1.44, 2.08)
High-risk group	6.25 (5.67, 6.90)	4.16 (3.72, 4.65)	4.29 (3.71, 4.95)	3.42 (2.91, 4.02)

Values are HRs with 95% CIs of medium- and high-risk groups compared with the low-risk group.

Abbreviations: aHR, adjusted hazard ratio; CI, confidence interval; Absence of inflammation, serum CRP level less than 1.0mg/dL; Presence of inflammation, serum CRP level 1.0 mg/dL or higher.

## Discussion

In this study, using large-scale cohort data, NRI was developed after identifying the main risk factors for one-year death, and with this risk score, the hemodialysis patients’ prognosis can be evaluated precisely. NRI included four nutritional factors in the criteria for PEW diagnosis[4]. To develop a risk index, a gold-standard outcome is necessary. In this study, one-year all-cause death, which is a hard outcome, was adopted as the gold-standard outcome. NRI showed a high accuracy in identifying high-risk patients. The prevalence of PEW in this study was 18.7%, which was consistent with those in previous studies[1–3, 7]. NRI may be useful for screening patients at increased risk of death due to malnutrition.

There have been various studies of the risk index for death on hemodialysis patients [8–10]. However, there has been no risk index for death developed on the basis from the data of nationwide large-scale Asian hemodialysis patients such as NRI. NRI is a new composite index of nutritional factors and represents complex nutritional conditions in hemodialysis patients. It has been reported that a composite index is more useful for identifying high-risk patients than a single index[8]. Moreover, evaluation of diverse nutritional conditions has been recommended[4]. Because NRI is composed of nutritional factors that are commonly measured in clinical settings, NRI would be useful for identifying high-risk patients for early treatment of their malnutrition.

The prevalence of PEW is higher in elderly dialysis patients than in younger patients[11, 12]. The serum albumin level in the elderly patients is lower than that in young patients[13]. Loss of appetite, dialysis-induced loss of serum albumin, comorbid conditions, and enhanced protein degradation are observed with aging and suggested to be the causes of hypoalbuminemia[14, 15]. This study showed that the cutoff serum albumin level was lower in the old group than in the young group. This suggests that hypoalbuminemia is a risk factor for death and the therapeutic target serum albumin level differs depending on age.

Reverse epidemiology has been reported as a specific pathological characteristics of hemodialysis patients[16]. A cohort study of Japanese hemodialysis patients showed that hypocholesterolemia is an independent predictor of death in hemodialysis patients[17]. In our study, the relationship between serum cholesterol level and the risk of death showed a U-shaped curve. This finding is similar to that of the cohort study, which also showed that low and high serum cholesterol levels are risk factors for death and that the main causes of death in hemodialysis patients with low and high serum cholesterol levels were infection and CVD, respectively[17]. In this study, two cutoff serum cholesterol levels were adopted in NRI, which may make NRI useful for predicting hemodialysis patients’ prognosis.

A U-shaped relationship between BMI and the risk of death has been reported, which is affected by hemodialysis patients’ characteristics such as age, inflammation, and comorbid conditions[18, 19]. In this study, the relationship was not U-shaped, which suggests that low BMI is a risk factor for death. Most of the subjects (74.2%) had BMIs lower than 23.0kg/m^2^. Considering the physical difference between Asians and Americans, a cutoff BMI of 20.0kg/m^2^ would be appropriate for hemodialysis patients.

Muscle mass is listed as a criterion for PEW [4]. However, muscle mass is not usually measured in Japanese dialysis facilities. In the paper on ISRNM’s PEW criteria, serum creatinine level measured before a hemodialysis treatment in maintenance hemodialysis patients was cited as an indirect measure of muscle mass[4]. Thus, in this study, serum creatinine level was measured as an alternative index of muscle mass. The distribution of muscle mass differs according to age and gender in dialysis patients[20]. Considering this finding, in this study, the cutoff serum creatinine level was determined according to age and gender.

nPCR has been used as an index of dietary protein intake in hemodialysis patients. In this study, no clear relationship between nPCR and risk of death was observed. The reasons for this finding might be as follows: nPCR is calculated from Kt/V and average BUN level, which shows some errors depending on Kt/V[21]. Moreover, nPCR is affected by residual renal function[22].

For the prevention and early detection of malnutrition, the nutritional status of patients should be monitored regularly, because early diagnosis and correction of malnutrition prevent clinical exacerbation[23]. NRI is useful for screening patients at risk of death with malnutrition. After the screening, detailed nutritional examinations are required in the patients identified to be at high risk of malnutrition. When malnutrition is diagnosed after ruling out or treating complications such as CVDs, inflammation, and cancers, nutrient intake should be improved by diet, oral supplements, and parenteral nutrition.

This study has several limitations. First, because of the observational nature of this study, the results may be biased by unmeasured confounders. Second, we did not examine the patients with missing data in this study, which might have caused selection bias. Third, the JRDR data did not include sufficient nutritional data on assessing malnutrition, comorbid conditions, and medications. Thus, we were unable to evaluate the effects of the differences in nutritional factors and medications such as statin on the risk of death. Further studies are needed to evaluate the relationship between these factors and NRI. Fourth, the differences in the baseline characteristics were observed between the risk groups. This might affect the relationship between the risk groups and the risk of death. However, considering the effects of baseline characteristics on the risk score, the cutoff levels were determined using multivariate Cox proportional hazards models adjusted for the baseline characteristics and ROC curves stratified by the baseline characteristics. The effects of the differences in the baseline characteristics were minimized.

## Conclusions

This study showed that the cutoff levels for hemodialysis patients were different from those in the criteria for PEW. NRI detects patients with malnutrition with a high-risk of death.
